# A “cat”-astrophic case of Bartonella infective endocarditis causing secondary cryoglobulinemia: a case report

**DOI:** 10.1186/s41927-022-00248-0

**Published:** 2022-03-25

**Authors:** Arani Vivekanantham, Rikesh Patel, Petra Jenkins, Gavin Cleary, David Porter, Fareed Khawaja, Eoghan McCarthy

**Affiliations:** 1 The Kellgren Centre of Rheumatology, Manchester Royal Infirmary, Oxford Road, Manchester, UK; 2 Centre for Epidemiology Versus Arthritis, University of Manchester, Manchester, UK; 3 Liverpool Heart and Chest Hospital NHS Foundation Trust, Thomas Drive, Liverpool, UK; 4 Alder Hey Children’s Hospital NHS Foundation Trust, Eaton Road, Liverpool, UK; 5 Nephrology Department, Manchester Royal Infirmary, Oxford Road, Manchester, UK; 6 NIHR Academic Clinical Fellow and Specialist Registrar in Rheumatology, Nuffield Department of Orthopaedics, Rheumatology and Musculoskeletal Sciences, Botnar Research Centre, Windmill Road, Oxford, OX3 7HD UK

**Keywords:** Bartonella endocarditis, Aspergillus, Culture-negative endocarditis, Cryoglobulinemia, Case report

## Abstract

**Background:**

Culture-negative infective endocarditis (IE) constitutes approximately 10% of all cases of IE. Bartonella endocarditis is a common cause of culture-negative endocarditis and is associated with a high mortality rate. To date, no cases of Bartonella IE has been reported in association with cryoglobulinemia in the UK. We present a unique case of Bartonella IE causing secondary cryoglobulinemia in a young female.

**Case presentation:**

A 17-year-old female with a background of pulmonary atresia and ventricular septal defect repaired with a cardiac conduit at the age of 4, presented with a one-year history of weight loss (from 53 to 39 kg) and poor appetite. She subsequently developed a vasculitic rash and haematoproteinuria with decline in renal function, requiring urgent hospital admission. Initial blood tests showed a near normal creatinine, but a raised cystatin C. Renal biopsy showed focal necrotizing glomerulonephritis with no acute tubular necrosis or chronic change. Subsequent blood tests supported a diagnosis of cryoglobulinaemic vasculitis (high rheumatoid factor, low complement, polyclonal gammopathy, Type 3 cryoglobulin). A weak positive PR3 meant there was some uncertainty about whether this could be a primary ANCA-associated vasculitis (AAV). Initial workup for an infectious cause, including multiple blood cultures, were negative. However, an echocardiogram showed definite vegetations on her surgical conduit. The patient did not respond to empirical antimicrobials and so was referred for surgical revision of her conduit. Tissue samples obtained intra-operatively demonstrated Bartonella species. With targeted antimicrobials post-operatively, she improved with resolution of immunologic abnormalities and at last review had a normal renal profile. On reviewing her social history, she had adopted several stray cats in the preceding year; and thus, the cause of the Bartonella infection was identified.

**Conclusion:**

This is the first reported case of Bartonella endocarditis causing secondary cryoglobulinemia reported in the UK. The key learning points from this case include that Bartonella endocarditis can present as a cryoglobulinaemic vasculitis and should be considered in any differential when the cause of cryoglobulinaemia is not clear and to enquire about relevant exposures especially when culture-negative endocarditis is suspected.

## Background

Culture-negative infective endocarditis constitutes approximately 10% of the total number of cases of infective endocarditis. Bartonella endocarditis is one of the most common causes of culture-negative endocarditis and is associated with a high mortality rate. Contact with animals, particularly cats, is a key risk factor. Other risk factors include pre-existing valvular disease, alcoholism, and homelessness [1, 2].

Cryoglobulinaemic vasculitis is a systemic vasculitis that can affect the small and medium-sized vessels. It is caused by the vascular deposition of circulating immune complexes of cryoprecipitating immunoglobulins. There are three main types of cryoglobulinaemia, as described in Table 1.

**Table 1:** Types of cryoglobulinaemia

Feature	Type I (monoclonal)	Type II (mixed)	Type III (mixed)
Frequency	25%	25%	50%
Cryoglobulin composition	Monoclonal IgM (sometimes IgG, IgA)	Combination of monoclonal (usually IgM) and polyclonal (usually IgG)	Polyclonal immunoglobulins IgG and IgM
Common causes	Lymphoproliferative diseases, multiple myeloma and Waldenström’s macroglobulinaemia	Hepatitis C virus infection	Hepatitis C virus infection, Sjögren’s syndrome, systemic lupus erythematosus, rheumatoid arthritis
Primary manifestations	Hyperviscosity ± thrombosis	Immune complex mediated vasculitis with multi-organ involvement	

Cryoglobulinaemic vasculitis classically presents with palpable purpura, asthenia and arthralgia and the clinical diagnosis must be confirmed by the detection of cryoglobulins [3, 4].

We present the first reported case of Bartonella infective endocarditis causing secondary cryoglobulinemia in the UK in a young female.

## Case presentation

A 17-year-old female college student presented to her GP with a one-year history of weight loss and poor appetite. She had a past medical history of pulmonary atresia and ventricular septal defect, surgically corrected at the age of 4 with a 18 mm Contegra conduit and was transitioning from paediatric to adult congenital cardiac care. She was initially suspected to have an eating disorder due to low mood and decreasing weight (from 53 to 39 kg). She subsequently developed a lower limb vasculitic rash. At that time urinalysis demonstrated haemoproteinuria (urine PCR 116 mg/mmol) resulting in an urgent renal referral. Her serum creatinine was 128 μmol/l (estimated glomerular filtration rate (eGFR) 58 ml/min/1.73 m2), however Cystatin C (which estimates GFR independent of muscle mass low muscle mass) estimated her GFR at 16 ml/min/1.73 m2 and accordingly she was admitted to hospital urgently for further urgent investigations.

On admission, she had a low body mass index (BMI 15) and was found to be pyrexial and have massive splenomegaly (19 cm). Initial immunological workup revealed a weakly positive ANCA PR3 (PR3 positive 2.3 (reference range 0–0.9)). She was commenced on a reducing regime of prednisolone on the second day of her admission empirically for presumed ANCA vasculitis, which resulted in a rapid resolution of the rash and improvement in her creatinine levels.

Rheumatology review was requested in view of a history of intermittent joint pains and ocular dryness. Blood tests were requested, the results of which are illustrated in Table 2.

**Table 2:** Additional blood test results

Blood test	Value with units (normal range)
Haemoglobin (Hb)	83 g/L (115–165)
Haematocrit (HCT)	0.266 Ratio (0.370–0.470)
Mean Cell Volume (MCV)	70 fl (80–90)
Mean Cell Haemoglobin (MCH)	21.7 pg (27.0–33.0)
Mean Cell Haemoglobin Conc. (MCHC)	312 g/L (320–365)
Red Blood Cells (RBC)	3.82 × 10*12/L (3.80–5.50)
White blood cells	3.6 × 10*9/L (4.0–11.0)
Neutrophils	2.67 × 10*9/L (1.80–7.50)
Lymphocytes	0.83 × 10*9/L (1.00–4.00)
Monocytes	0.12 × 10*9/L (0.20–1.00)
Basophils	0.02 × 10*9/L (0.20–1.00)
Platelets	218 × 10*9/L (150–400)
Rheumatoid factor (RhF)	211 (positive)
Anti–cyclic citrullinated peptide (anti-CCP)	< 1 (negative)
Anti-nuclear antibody	Negative
Extractable nuclear antigen (ENA) profile	Negative
Complement level C3	0.90 g/L (normal)
Complement level C4	0.09 g/L (low)
Immunoglobulin G (IGG)	45.05 g/L (raised)
Immunoglobulin A (IGA)	0.88 g/L (normal)
Immunoglobulin M (IGM)	3.25 g/L (raised)
AP100 Alternative Pathway Haemolytic Complement	50 (normal)
Beta-2 glycoprotein I (β2GP1) antibody	Negative
Cardiolipin antibody	Negative
Lupus anticoagulant	Negative
Prothrombin time (PT)	10.6 (normal)
Activated partial thromboplastin time (aPTT))	29.1 (normal)
Dilute Russell viper venom time (DRVVT) screen ratio	0.98 (normal)
Anti-phospholipase A2 receptor (PLA2R) antibodies	< 3 (normal)
Anti–glomerular basement membrane (anti-GBM) antibody	< 0.2 AI (normal)

**Fig. 1:**
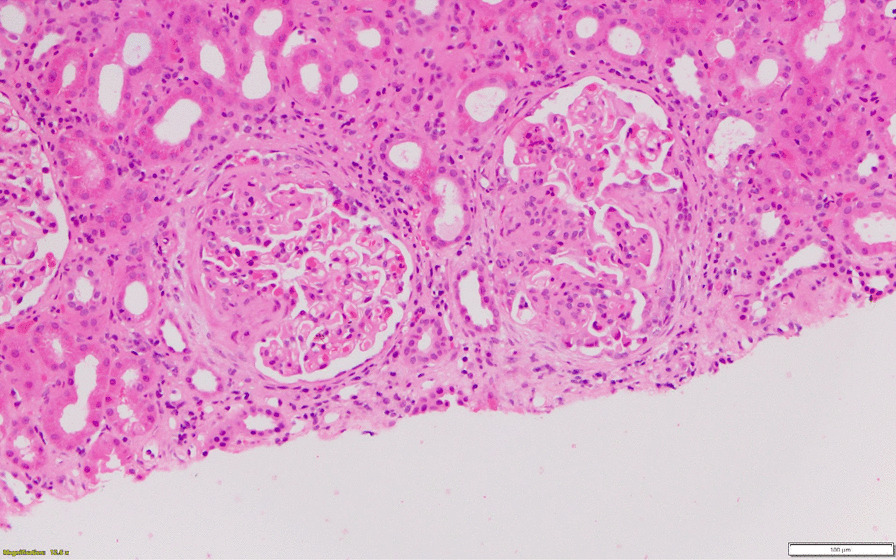
Histopathology image of renal biopsy. Glomeruli showing segmental lesions with proliferation and crescents

A renal biopsy (Fig. 1) showed focal segmental proliferative and necrotising lesions with double contours and fibrocellular crescents. Tubulointerstitium was normal with no acute tubular necrosis or chronic changes. Immunofluorescence showed C3 (3 +), Lambda (2 +), IgM (1 +), C1q (+ / −). Immune complexes were seen in paramesangial, mesangial and subepithelial locations. These biopsy features were in keeping with an infection related membranoproliferative glomerulonephritis (Type 1) pattern.

Given the clinical features, renal biopsy findings and immunological abnormalities a potential diagnosis of cryoglobulinaemic, rather than ANCA vasculitis, was made with further testing confirming a Type 3 cryoglobulin (polyclonal IgG). Serial blood cultures were negative and initial investigations for an infectious cause (e.g., Hepatitis B, C serology and HIV antibody screen) were also negative. Bone marrow biopsy showed reactive changes and excluded haematological malignancy.

**Fig. 2:**
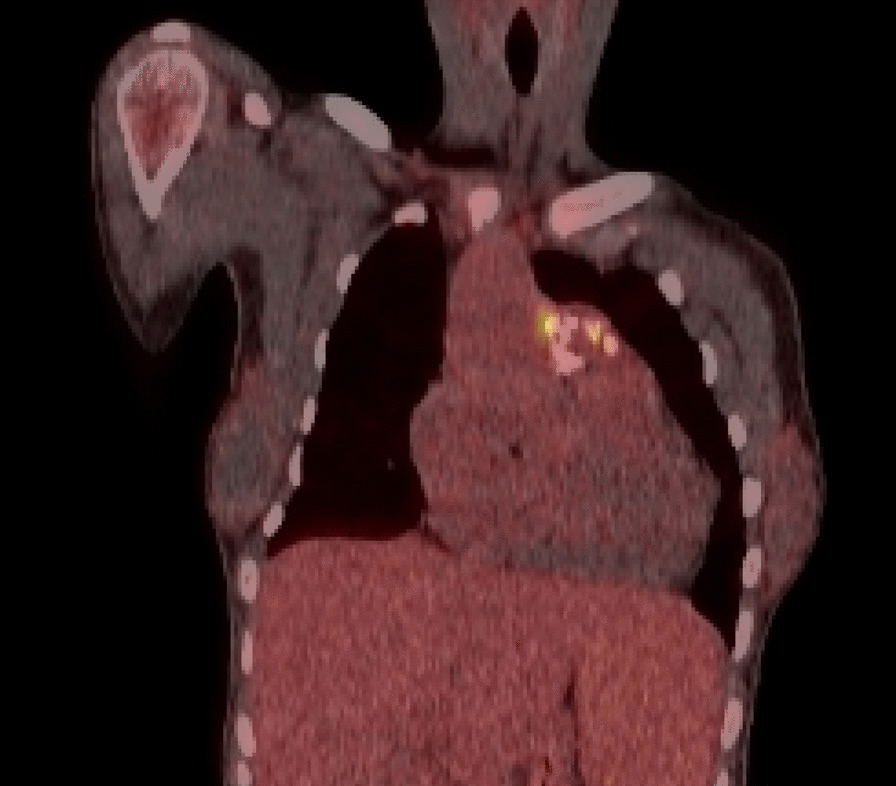
PET scan image showing uptake around the conduit valve which is in the pulmonary position. Written consent to publish this image was obtained from the patient

Additional investigations to identify an infective cause resulted in an echocardiogram showing vegetations on her conduit. A positron emission tomography (PET) scan was then requested as the changes seen on the conduit could have been due to conduit degeneration, which is common after 13 years. The PET scan (Fig. 2) showed uptake around the conduit valve in the pulmonary position supporting an infectious or inflammatory aetiology, with a CT pulmonary angiography (CTPA) demonstrating septic emboli in the anterior basal right lower lobe.

Empirical antibiotics treatment for endocarditis (vancomycin, gentamycin, and rifampicin) were commenced. Inflammatory markers and renal function failed to improve following two weeks of intravenous antibiotics for bacterial endocarditis and accordingly the patient was referred for surgical revision of her conduit. This was performed at a tertiary paediatric cardiothoracic unit. Tissue samples cultured positive for Bartonella and aspergillus species. Antibiotic therapy was altered to doxycycline and anti-fungal therapy whilst oral steroids were continued. Following completion of antibiotic treatment and steroid taper, she achieved resolution of her cryoglobulinaemia and normalization of renal function, RhF and C4.

The unifying, final diagnosis was, therefore, Type 3 Cryoglobulinaemia with crescentic glomerulonephritis secondary to Bartonella endocarditis + / − fungal infection of conduit. On reviewing the social history, the patient’s mother revealed that she had adopted several stray cats in the preceding year; this was identified as the cause of the Bartonella infection.

The patient was subsequently reviewed in the Rheumatology outpatient clinic, approximately six months following her initial admission. She was found to be progressing well; her splenomegaly had resolved, appetite improved, she had regained weight and her antibiotics and steroids have been stopped. Her biochemical markers have also improved; with creatinine of 51 μmol/l (from 128, peak creatinine 192 μmol/l), CKD-EPI-eGFR > 90 ml/min/1.73m2 (from 16) and her most recent cryoglobulin remains negative. Her post-operative follow up in the adult congenital cardiac clinic has shown an excellent outcome. She has returned to college, however, has continued to house stray cats at home despite medical advice.

## Discussion and conclusion

In this report we describe a rare presentation of cryoglobulinaemia, and renal impairment in a patient with Bartonella endocarditis.

Bartonella species are small Gram-negative bacilli. The first cases of Bartonella causing infective endocarditis were reported in two separate reports in 1993 [3, 4] and patients typically present similar to that of subacute bacterial endocarditis caused by other bacteria [5]. Bartonella is now considered one of the most common causes of culture-negative endocarditis and the number of cases reported in the literature have been increasing worldwide. The mortality rate in one retrospective study of 101 patients diagnosed with Bartonella endocarditis was found to be 12% [6] which may relate to the difficulty in diagnosis as the organism is difficult to isolate using standard microbiologic culture techniques [5].

Patients with infective endocarditis (IE) irrespective of cause can develop several forms of kidney disease: an infection-related immune complex-mediated glomerulonephritis (GN), renal infarction from septic emboli, a drug-induced acute interstitial nephritis or acute tubular necrosis. One study found that 45% of patients with *Bartonella* endocarditis have kidney failure [6, 7].

Endocarditis-associated glomerulonephritis can show significant variability in histopathologic appearance with both immune complex–mediated and pauci-immune glomerulonephritis described [6]. Our case is notable as suggests a potential immunological mechanism for the renal injury observed in Bartonella endocarditis via type 3 cryoglobulinaemia. We postulate that the chronic Bartonella infection resulted in polyclonal B cell activation resulting in hyper‐γ‐globulinemia. Whilst this response may be beneficial for early host defence by generating antibodies specific to Bartonella it may also have a deleterious longer-term effect during chronic infection by potentially augmenting anti‐self‐responses resulting in automimmunity and in our case cryoglobulin production. There has only been one other case report of Bartonella infective endocarditis complicated by cryoglobulinemia and renal failure reported in a middle-aged male. There were similarities between the two cases: the presence of a Type 3 mixed cryoglobulin associated with an acute kidney injury with haemoproteinuria in the setting of negative blood cultures [7], a background of cardiac surgery (bioprosthetic aortic valve replacement) and exposure to cats. However, the initial presentations of both cases were different; our patient presented insidiously over a year whereas in the case previously described the patient presented acutely. Moreover, the patient was ANCA negative in the previous case making our case unique.

There are case reports of bartonella infective endocarditis with ANCA and glomerulonephritis reported [8]. Unlike these and the case described above, which had bartonella IE and cryoglobulins, our case had both cryoglobulins and PR3 positivity.

One of the differentials in our patient was an ANCA associated vasculitis given her weakly positive PR3. Positive ANCA/PR3 testing has been reported in some cases of IE, which may lead to diagnostic confusion as in our case. Bartonella endocarditis appears to be associated with particularly high rates of ANCA/PR3 positivity. In a literature review of 54 cases of Bartonella IE-associated glomerulonephritis, 78 percent were ANCA positive by indirect immunofluorescence and/or enzyme-linked immunosorbent assay (ELISA), and 67 percent were positive for proteinase 3 (PR3) [9]. ANCA associated vasculitides are rare, potentially life and organ threatening diseases in children and young people. However, the sensitivity and positive predictive value of a positive ANCA is poor, particularly in low titres, with a retrospective case series identifying that ANCA testing has a positive predictive value of 54% for diagnosis and concluded that a positive result is not a definitive diagnostic indicator of ANCA-associated vasculitis [10]. Expert correlation of the presence of ANCA antibodies with clinical features is needed to confirm a diagnosis of ANCA associated vasculitis.

In terms of the management, our patient and the case reported in the literature were initially treated with steroids and antibiotics empirically. In the previously reported case, the patient underwent additional plasmapheresis as well as required intermittent haemodialysis. Plasmapheresis was considered in our case but deferred as her renal function improved with the introduction of steroids and correct antimicrobial treatment once a unifying diagnosis was made. Rituximab was also considered but given the requirement for conduit revision surgery, held.

Our case describes the association between Bartonella infection and Type 3 cryoglobulinemia and highlights a potential important immune-mediated mechanism for renal injury in such cases. In patients with an unexplained cryoglobulinaemia assessment for Bartonella infection may reveal an under-recognised cause. As cryoglobulin related disease is relative rare in children and young people, patients presenting with Bartonella, and renal impairment would benefit from testing for cryoglobulins to allow early recognition and allow informed management decisions.

We acknowledge that infection in isolation can cause many of the features seen in this case but given the degree of immune activation seen with the low complement, positive RhF, and Type 3 cryoglobulinaemia we feel it is more likely that the infection triggered an aberrant immune response resulting in cryoglobulin production and thus initial immunosuppression and antibiotic/ fungal treatment were both indicated. Moreover, a Type 3 cryoglobulin can be part of an autoimmune process [11].

In summary, this is the first case of Bartonella endocarditis causing a secondary cryoglobulinemia and renal failure reported in the UK. There are a number of key learning points from this case which include that Bartonella endocarditis can present as a cryoglobulinaemic vasculitis and should be considered in any differential when the cause of cryoglobulinaemia is not clear, patients with intracardiac surgical repairs/tissue have high risk of IE therefore high index of suspicion is required in patients presenting with systemic symptoms/vasculitis, complex multi-modality cardiac imaging is helpful in evaluating congenital cardiac IE and the importance of enquiring about relevant exposure especially when culture-negative endocarditis is suspected.

## Data Availability

All data generated or analysed during this study are included in this published article.

## Abbreviations

PCR: Polymerase chain reaction
eGFR: Estimated glomerular filtration rate
GP: General practitioner
BMI: Body Mass Index
IE: Infective endocarditis
